# Leaching and Geochemical Modelling of an Electric Arc Furnace (EAF) and Ladle Slag Heap

**DOI:** 10.3390/toxics10010010

**Published:** 2022-01-01

**Authors:** Mojca Loncnar, Ana Mladenovič, Vesna Zalar Serjun, Marija Zupančič, Hans A. van der Sloot

**Affiliations:** 1 SIJ Acroni d.o.o., C. Borisa Kidriča 44, 4270 Jesenice, Slovenia; 2 Slovenian National Building and Civil Engineering Institute, Dimičeva u. 12, 1000 Ljubljana, Slovenia; ana.mladenovic@zag.si (A.M.); vesna.zalar@zag.si (V.Z.S.); 3 Faculty of Chemistry and Chemical Technology, University of Ljubljana, Večna pot 113, 1000 Ljubljana, Slovenia; Marija.Zupancic@fkkt.uni-lj.si; 4 Hans van der Sloot Consultancy, Glenn Millerhof 29, 1628TS Hoorn, The Netherlands; hans@vanderslootconsultancy.nl

**Keywords:** EAF slag, field verification, geochemical modelling, ladle slag, leaching, release prediction, steel slag heap

## Abstract

Old metallurgical dumps across Europe represent a loss of valuable land and a potential threat to the environment, especially to groundwater (GW). The Javornik electric arc furnace (EAF) and ladle slag heap, situated in Slovenia, was investigated in this study. The environmental impact of the slag heap was evaluated by combining leaching characterization tests of landfill samples and geochemical modelling. It was shown that throughout the landfill the same minerals and sorptive phases control the leaching of elements of potential concern, despite variations in chemical composition. Although carbonation of the disposed steel slags occurred (molar ratio CO_3_/(Ca+Mg) = 0.53) relative to fresh slag, it had a limited effect on the leaching behaviour of elements of potential concern. The leaching from the slag heaps had also a limited effect on the quality of the GW. A site-specific case, however, was that leachates from the slag heap were strongly diluted, since a rapid flow of GW fed from the nearby Sava River was observed in the landfill area. The sampling and testing approach applied provides a basis for assessing the long-term impact of release and is a good starting point for evaluating future management options, including beneficial uses for this type of slag.

## 1. Introduction

According to estimates, there are between 150,000 and 500,000 historic and active landfills in Europe, many of which are located in urban and semi-urban environments [1,2,3]. Among others, inorganic wastes, such as mined waste rock, low grade stockpiles, tailings and metallurgical slag have previously been disposed in these landfills [4]. Although in the last few decades metallurgical slag, especially that coming from ironmaking and steelmaking, have been widely used in various fields, e.g., cement production, road building and restoration of the marine environment [5], many old metallurgical dumps still exist across Europe. Such heaps represent a loss of valuable land as well as a potential threat to the environment, especially to groundwater. Many factors affect the geochemical behaviour of leachates from disposed alkaline slag, including the chemistry of the infiltrating water, the physicochemical composition and age of the residue, the nature of any co-deposited wastes, the hydrogeological setting (e.g., flow rates, redox status, residence time of water), the properties of the native ground, and the receiving water chemistry [6,7]. The impacts of alkaline leachates can include an increased water pH, a high chemical oxygen demand and oxygen depletion in the water column, high sulphate loadings, salinity, and an increase in the concentrations of metals [7,8]. Since metallurgical heaps are often heterogeneous in terms of their resource content, a holistic approach, a broad knowledge base and considerable experience are needed to gain sufficient knowledge regarding their environmental properties.

In recent years the EU commission has placed a strong emphasis on a transition to a resource-efficient, clean and circular economy [9,10]. Companies will need to use waste as a resource, transforming end-of-life products into new raw materials for production, also taking into consideration landfill mining. The valorisation of historical and future waste streams through both waste-to-material (WtM) and waste-to-energy (WtE) practices is proposed by the concept of Enhanced Landfill Mining (ELFM) [3,11]. However, one of the main concerns that hinders the more extensive recycling of industrial by-products and waste and old metallurgical heaps is insufficient knowledge about their environmental properties. In recent years standardized characterization leaching tests (e.g., according to EN 14429, EN 14405 and the analogous US EPA LEAF methods such as EPA 1313 and EPA 1314) have provided a good basis for evaluating the environmental aspects, especially for applications involving the beneficial use of industrial secondary raw materials [12,13,14,15]. In the past, studies assessing the environmental impact of old metallurgical dumps, especially old steel dumps, have focused on the geochemistry of the alkaline leachates to the groundwater environment and surface water environment [7,16,17,18]. The long-term evolution of leachates at large disposal sites under ambient environmental conditions has also been described recently [19]. Nowadays geochemical modelling plays an important role in evaluating the quality of water from landfill and metal speciation within soil in an industrial area [15,20,21]. Recently it was shown that investigating the release of heavy metals from old metallurgical dumps under varying pH conditions can be an appropriate approach to evaluate different management scenarios [22,23]. The tools that combine characterization leaching tests with chemical speciation modelling in order to assess environmental leaching [15] have not yet been applied to assess the environmental impact of electric arc furnace (EAF)/ladle slag heaps.

The main objectives of the present study were to assess the long-term environmental impact of the Javornik EAF/ladle slag heap, Slovenia, on the local environment and GW, to provide input with regard to the next steps required to close the landfill, and to assess the potential for beneficial use of the slag. Approximately 400,000 tonnes of steel slags are deposited there, primarily consisting of EAF slags resulting from the production of carbon and stainless steel, alongside smaller quantities of ladle slags and other steelmaking wastes. The slag heap is in the process of being closed. In order to assess the phases release and their role in long-term leaching from the slag deposits, a pH dependence leaching test was applied on a composite of individual field samples from different locations and depths, followed by geochemical speciation modelling using LeachXS^TM^-Orchestra [21]. This follows the recommendation by the US EPA Leaching Environmental Assessment Framework (LEAF) [15], which represents a considerable shift in the methodology for leaching assessment away from current approaches, are typically based on single-batch tests and do not necessarily reflect realistic exposure conditions. Prior experience in modelling alkaline by-products was helpful in this respect [15,24,25,26]. A detailed mineralogical characterization of the composite sample was performed to allow a comparison with the leaching-controlling phases identified. A batch extraction test of individual landfill samples and local GW monitoring data are placed in context with the pH dependence leaching test results from the composite landfill material in order to obtain an insight into spatial variations in the release of contaminants from the EAF/ladle slag heap, the long-term effects of changing the conditions of exposure, and the potential for its beneficial use.

## 2. Materials and Methods

### 2.1. Sampling Slag from the Metallurgical Slag Heap

**Figure 1:**
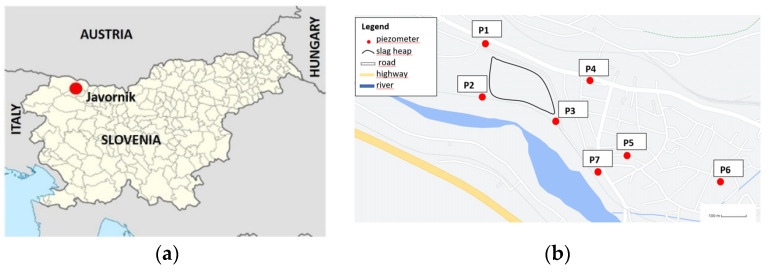
(**a**) Location of the landfill site in the town of Jesenice in NW Slovenia; (**b**) locations of the 7 piezometers for GW monitoring, marked by yellow rectangles labelled P1-P7.

The Javornik steel slag heap is located in the town of Jesenice (NW Slovenia; X:142,982 Y:429,595; Figure 1), which is known for its long history of mining and ironworks dating back to the 14th century. Approximately 400,000 tonnes of metallurgical waste are deposited here, primarily consisting of steelmaking slags from the production of carbon and stainless steel. The heap is approximately 3 ha large with a landfill area of 1.95 ha.

Sampling was conducted by an accredited laboratory, according to SIST EN 14899:2006 (Characterization of waste—Sampling of waste materials—Framework for the preparation and application of a Sampling Plan). Sampling points were selected systematically in the correct raster to ensure coverage of the entire landfill. Eleven samples were sampled in total, with 6 samples taken from a depth of approximately 200 mm below the landfill surface (designated as L1–L6), and 5 samples from a depth of around 6 m (designated as L7–L11) (Table 1). A composite landfill sample, designated PTO, was made from a mixture of 10 of the individual samples (L2–L6 and L7–L11).

The bulk chemical composition was defined for the composite landfill sample PTO, as well as for the individual landfill samples (L1–L11), while the phase composition was investigated only for the composite landfill sample PTO. The individual landfill samples (L1–L11) were subjected to a single-step batch leaching test (EN 12457-4, 2002), whereas the composite landfill sample PTO was subjected to a laboratory characterization leaching test (pH dependence leaching test (SIST EN 14429:2015)).

### 2.2. Groundwater (GW) Monitoring (2011–2019)

Starting in 2011, GW monitoring was carried out twice annually (usually in spring and autumn), using 7 piezometers (P1–P7; Figure 1b). The monitoring was performed by an accredited laboratory, according to SIST ISO 5667-11:2010. Piezometers P1 and P2 were located outside of the area occupied by the EAF/ladle slag heap, and thus represented reference piezometers. The P1 piezometer (X:143,216.82 Y:429,369.19 Z:539.40) is located on the northern edge of the slag heap, with the aim of observing the level of groundwater that flows into this area. The P2 piezometer (X:143,014.16, Y:429,376.19, Z:543.50) is located on the upper part of the slag heap, in the direction of the Sava River. With this piezometer it is possible to determine the inflow or supply of GW from the direction of the river, where the highest inflows are expected during periods of high water. Piezometer 3 (X:142,881.10, Y:429,659.46) is located on the south-eastern edge of the slag heap. It represents the place where the GW flows out from beneath the landfill, and the chemical analysis of this GW will directly show the effects of the slag heap on the GW. By comparing the analysis of all three, of course all taken on the same day, it will be possible to determine the impact of the Sava River, the hinterland waters from the north, and the impact of the steel slag heap on the surrounding area. An additional four piezometers were constructed in the intended target zone to take into the consideration the wider area that may be affected by the EAF and ladle slag (P4 (X:143,083.4, Y:429,733.0), P5 (X:142,795.4, Y:429,859.0), P6 (X:142,710.9, Y:430,135.2) and P7 (X:142,752.25, Y:429,767.4). The piezometer P7 is not so representative, however, since it is at a depth of 5 m, while all the others are located at a depth of 20 m. GW monitoring is still being carried out. The GW monitoring data taken into consideration in this study was from the period between 2011 and 2019.

### 2.3. Chemical Analysis

#### 2.3.1. Bulk Chemical Composition

The bulk chemical composition of the major elements in the individual slag samples (L1-L11) and the composite landfill sample PTO were determined by XRF (MAGI’X FAST, PANalytical), and those of the minor elements by ICP-MS (Agilent 7500ce, Agilent Technologies Inc., Santa Clara, CA, USA) following acid digestion (first step: 3 mL H_3_PO_4_ s. p. (Fluka) + 5 mL HNO_3_ s. p. (Fluka) + 1 mL HF s. p. (Fluka); second step: 6 mL H_3_BO_3_ (Sigma-Aldrich) + 2 mL HNO_3_ s. p. (Fluka)). The C and S contents were measured by carbon/sulfur analyser (CS 233, LECO). All the analyses were performed in duplicate.

#### 2.3.2. Characterization Leaching Tests

The individual landfill samples (L1-L11) were subjected to a single step batch leaching test (EN 12457-4, 2002; d < 10 mm), whereas the composite landfill sample PTO was subjected to a pH dependence leaching test (SIST EN 14429:2015; d < 1 mm). All the analyses were performed in triplicate.

#### 2.3.3. Leachates Analysis

pH (SIST ISO 10523:2008; pH/Conductivity meter SevenMulti, Mettler Toledo), Eh (SIST DIN 38404-6:2000; T 90-MB, Mettler Toledo), EC (SIST EN 27888:1998; pH/Conductivity meter SevenMulti, Mettler Toledo) and Cr(VI) concentrations (SIST ISO 11083:1996; Cary 1E (UV VIS), Varian) were determined in all of the batch leachate and pH-dependent leachate samples. The metals were analysed by either ICP-MS (SIST EN ISO 17294-2, 2005; Agilent 7500ce, Agilent Technologies Inc.) or ICP-OES (ISO 11885:2007, ICP-OES OPTIMA 2000 DV, Perkin Elmer). Additionally, the anion content (Cl^−^, F^−^, SO_4_^2^^^−^^, NO_3_^−^) (ISO 10304-1:2007; IC, 761 COMPACT IC, Metrohm) and DOC (SIST ISO 8245:2000; Multi N/C 2100 S (CHD), Analytik Jena)) were determined.

#### 2.3.4. GW Analysis

In addition to pH (SIST ISO 10523:2008; pH meter Mettler Toledo), ELP (SIST EN 27888:1998, WTW universal multi-parameter portable meter), and Eh (DIN 38404-6, WTW universal multi-parameter portable meter), which were measured in the field, the content of metals (SIST EN ISO 17294-2:2017, ICP-MS 7800 Agilent), anions (Cl^−^, F^−^, SO_4_^2^^−^, NO_3_^−^) (ISO 1034-1:2007, IC Dionex), K (FAAS, CVAAS-Perkin Elmer), TOC (SIST ISO 8245:2000, Shimadzu), AOX (ISO 9562:2000, AOX analyser Euroglas), NH_4_^+^ (SIST ISO 7150-1:1996; UV/VIS spectrometer HACH), HCO_3_^−^ (titrator-Metrohm), NO_2_^−^ (SIST EN 26777:1996, UV/VIS spectrometer HACH), CN^−^ (SIST EN ISO 14403-2:2013; CFA) and PAH (SIST EN ISO 17993:2004 modif.; GC MS/ECD Agilent) were also determined in all the GW samples.

### 2.4. Mineralogical Characterization

#### 2.4.1. X-ray Diffraction (XRD) Analysis

The mineral phases of the composite landfill sample PTO were characterized by X-ray diffraction (XRD) using a PANalytical Empyerian diffractometer (PANalytical B.V., Almelo, The Netherlands) in Cu Ka1 configuration. Data were collected at room temperature over a 2Θ range from 5° to 70°, using scanning steps of 0.01°2Θ and a measurement time of 150 s per step. X’Pert HighScore Plus software (PANalytical B.V. Almelo, The Netherlands) was used to identify the crystalline phases of the samples investigated, with the Powder Diffraction File PDF-4+ (ICDD, Newtown Square, PA, USA) database used as a source of reference data. The composite sample was ground in an agate mortar to a particle size of less than 40 μm. The sample was mounted in a 27 mm diameter sample holder using the backfilling technique.

#### 2.4.2. Scanning Electron Microscopy (SEM) Analysis

The morphological, microstructural, and semiquantitative chemical properties were investigated on polished cross sections of the composite landfill sample PTO via scanning electron microscopy (SEM) using a JEOL JSM IT500 (Tokyo, Japan) microscope equipped with energy dispersive spectroscopy EDS (Oxford instruments, Bognor Regis, UK). The low-vacuum mode was used, with a chamber pressure of 90–100 Pa and an accelerating voltage of 20 kV.

### 2.5. Data Comparison and Geochemical Modelling

The LeachXS^TM^ database/expert system was used for the management of data, i.e., the pH dependent leaching data, batch data, GW data, and data for visualizing the calculated and measured results [27].

Chemical speciation of the eluates from the pH dependence test (SIST EN 14429:2015) was calculated using the ORCHESTRA modelling framework [28] embedded in LeachXS^TM^ [29]. The input to the model consists of fixed element content availability (i.e., the amount of each element present that can participate in liquid-solid equilibrium partitioning), as obtained from the maximum amount leached in the pH dependent leaching test over a pH range of 1.5–13. Aqueous speciation reactions and selected mineral precipitates were taken from the MINTEQA2 database and supplemented with data from the literature as a data patch. Ion adsorption onto organic matter was calculated by the NICA-Donnan model [30], using the generic adsorption reactions for all elements as published by Milne et al. [31,32], starting from a description of the DOC concentration as a function of pH (fitting a polynomial curve to the results of the pH dependence leaching test), and correcting for the reactive part of the DOC (DHA-dissolved humic acid fraction). The adsorption of ions onto iron and aluminium oxides was modelled according to the generalized two-layer model by Dzombak and Morel [33], starting from the fraction of Fe content available, and calibrating against the measured release of As and V. The total carbonate in the sample is used as the input and calibrated against the measured release of Ca.

The inputs to the model consisted of the availability of the elements, selected minerals that could potentially control solubility, the ettringite substitution reactions, and the concentrations of reactive Fe-(hydr)oxides (HFO). HFO was quantified as 10% of the Fe fraction available (not all soluble Fe is available as a reactive surface), and clay as 10% of the weight fraction of d < 63 µm (not all of the clay mass is available for sorption). Based on the results of preliminary modelling, where the redox state (the sum of pH and pE) was quantified as 12 [34], the redox state was quantified as 14 in the model runs reported. With slightly more oxidative conditions a better fit was achieved for the pH dependent data measured, and the predictive capacity of the model was improved. Additionally, other model runs were performed considering differing carbonate levels (CO_3_/(Ca+Mg) = 0.53, 0.84 and 0.96) in order to better understand the carbonation of the individual slag samples (L3, L4, L7 and L11).

The results of the multi-element geochemical modelling are presented in graphs showing both the measured versus modelled concentrations and the distribution between the speciation calculated in the solid matrix (the minerals and the sorption to the clay and Fe-oxides) and the solution (free and inorganic complexes), adding up to the available content. Model runs were carried out at both L/S = 10 (test conditions) and L/S = 0.4 (pore water simulation), with all other parameters remaining the same. Both a wide pH range and a wide L/S range were included, so that a wide range of possible scenarios were addressed with respect to the conditions of exposure. The low L/S of 0.4 corresponds to the pore water conditions, but also allows for identification of the conditions controlling solubility (sections where the high and low L/S curves overlap).

The selection of mineral phases that were able to precipitate was based on prior experience with steelmaking slags [24,25,26]. Selection of the most likely and relevant phases was made based on the degree of fit over a wider pH range, and expert judgment concerning the likelihood that minerals could occur in such a system. At the same time, the number of minerals selected was reduced by selecting only those which represented at least one thousandth of the amount of the element available in the system.

The database/expert system LeachXS^TM^ [29] was used for data management of the pH dependent leaching data, the single step batch leaching data from individual site samples, and the GW data for visualizing both the calculated and measured results.

## 3. Results

### 3.1. Chemical Characterization of the Landfill Samples

Chemical characterization of the composite landfill sample PTO revealed that the heap is primarily composed of oxides that mainly occur in steelmaking slags (e.g., CaO, SiO_2_, MgO and Al_2_O_3_) (Table 1). With respect to its chemical composition, the content of each element fell within the range of values reported in the literature for the chemical composition of steelmaking slags, as detailed in Table 2.

Some variation was observed between the individual landfill samples sampled at approximately 200 mm below the surface of the heap and those sampled at a depth of around 6 m. On average, samples L1–L6 have higher contents of CaO, Cr_2_O_3_ and carbon than samples L7–L11. The higher carbon content indicates a more pronounced carbonation of the material near the surface of the EAF and ladle slag heap. Samples L7–L11 have a higher SiO_2_ content than samples L1–L6. The Fe content in sample L3 was 30.5 wt.%, twice as high as the Fe content in other samples, so was therefore not taken into consideration in Table 3. A high MnO content was also observed in L1, with a value of 12.4%, and was not considered in Table 3. The MnO content in samples L7–L11 varied a lot, ranging from 0.9 wt.% to 3.3 wt.%.

Variations between the chemical compositions of the individual landfill samples sampled from around 200 mm below the surface of the heap and those sampled at a depth of around 6 m may be due to the fact that different mixtures of slag types with different chemical compositions were disposed at the landfill during its period of operation. When disposal began (in 1987), EAF carbon slag (EAF C slag) was disposed at the landfill, among others, while later, when the company introduced the processing of EAF C slag for use in construction, mainly EAF stainless slag (EAF S slag) and ladle slag were disposed there.

### 3.2. Mineralogical Characterization

**Figure 2:**
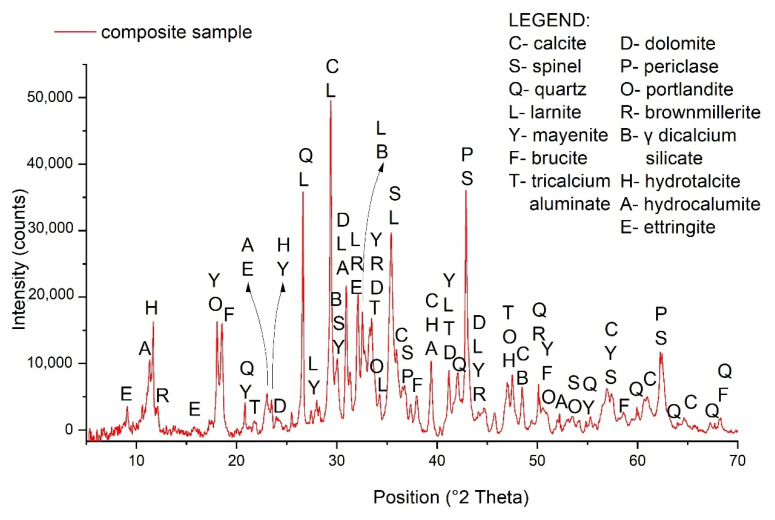
XRD pattern of the composite landfill sample PTO.

Detailed mineralogical characterization (XRD and SEM/EDS analysis) was conducted on the composite landfill sample PTO in order to confirm that steelmaking slags are the predominant material in the landfill. Although the X–ray diffraction (XRD) pattern of the PTO was, in general, very complex, showing several mineral phases, the minerals identified were similar to those frequently found in steelmaking slags. As can be seen from Figure 2, the main minerals observed in the composite landfill sample PTO were calcite, spinel, dicalcium silicate (larnite, γ dicalcium silicate), calcium aluminates (mayenite, tricalcium aluminate), hydroxides (brucite, portlandite), dolomite, periclase and brownmillerite.

**Figure 3:**
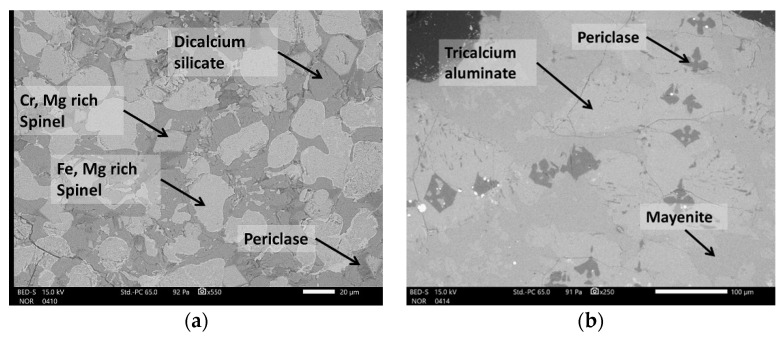
General SEM micrographs of the slag grain in the PTO sample, composed of dicalcium silicate, spinels and periclase (**a**), and a grain composed of tricalcium aluminate, mayenite and periclase (**b**).

**Figure 4:**
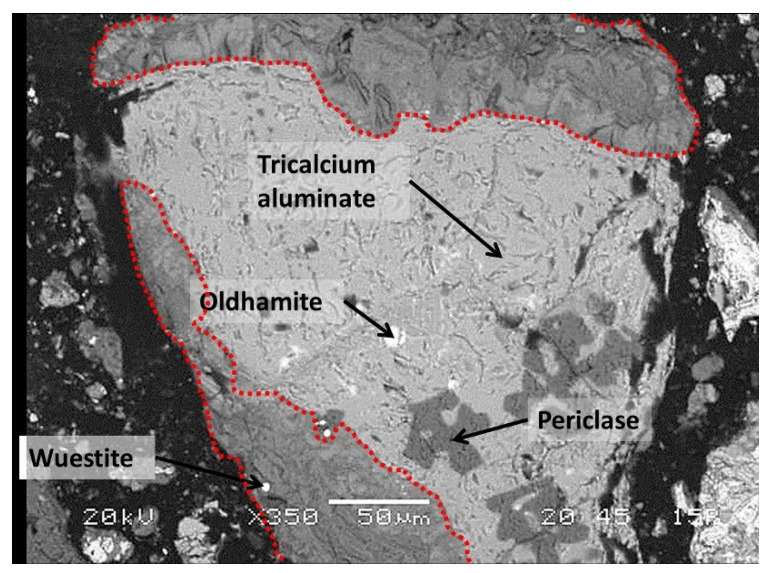
A general SEM micrograph of the prehydrated slag grain with the constituent phases defined by EDS, and the reaction rim marked with a red dotted line.

The mineral phases identified by XRD were also confirmed by SEM analysis (Figure 3). Spinels occurred in two forms, with the majority of spinels enriched with Fe and Mn (anhedral to subhedral in form, lighter, often quite heterogeneous) (Figure 3a). Less commonly, spinels were enriched with Cr and Mg (euhedral in shape, smaller crystallites, darker) (Figure 3a). The dicalcium silicate occurred in a variety of forms, but mostly in the form of round-shaped grains (Figure 3a). Similar, C_3_A also occurred in more or less round-shaped grains. Mayenite is xenomorphic and presents a mineral matrix of tricalcium aluminate (Figure 3b). Periclase was evenly distributed across the composite landfill sample PTO. The phases’ wuestite and oldhamite were also identified in traces by SEM/EDS (Figure 4).

The mineral phases identified are in line with the mineral phases reported in the literature. Spinels ((Mg, Al, Fe, Cr)_2_O_4_), larnite, periclase, and brownmillerite are minerals commonly found in EAF C slag [36,37,38,39]. In ladle slag dicalcium silicate (γ–C2S), larnite, mayenite and tricalcium aluminate are more frequently observed [38,40,41,42]. Larnite and mayenite, however, may also be found in EAF slag [35,36,43]. The quartz most likely originates from other materials (sand, refractory brackets) that were probably also dumped on the metallurgical slag heap. Herbeline et al. (2020) also concluded that quartz found in an old dump, where steel slags had accumulated over six decades, came from other materials stored with the slag on-site (e.g., sand and refractory bricks) [44]. A mineralogical comparison between the composite landfill sample PTO and “fresh” steelmaking slags (EAF S slag and ladle slag) coming from the same steel company, sampled just after the steelmaking process [24,45], revealed similar mineral phases. In the “fresh” EAF S slag and ladle slag the brownmillerite was not, however, observed, due to the fact that EAF C slag was not investigated in those studies. It is well known that brownmillerite is a constituent of EAF C slag [36].

It can be concluded from the mineralogical investigation (XRD and SEM/EDS) that the various types of steelmaking slag (i.e., EAF C slag, EAF S slag and ladle slag) were the main materials dumped at the Javornik EAF/ladle slag heap.

**Figure 5:**
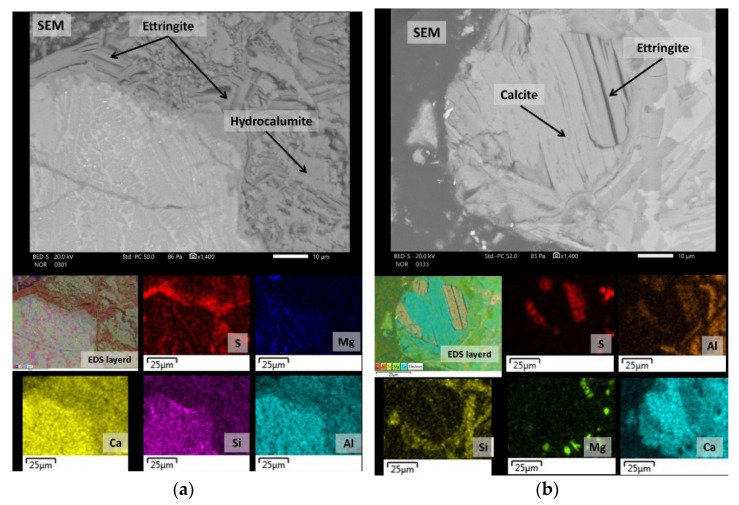
Results of the SEM/EDS mapping analysis of the slag grain reaction rim, composed of (**a**) two different CAH phases, and (**b**) calcite and ettringite.

Secondly, mineralogical characterization was used to observe the mineral phases suggested by the geochemical modelling as phases that dictate the leaching behaviour at a material’s natural pH (see Section 3.4, ‘Geochemical modelling’). Calcite, portlandite, brucite, ettringite, hydrocalumite and hydrotalcite were assigned as secondary mineral phases (identified by XRD and SEM/EDS). A previous study [24] has shown that, to a large extent, the secondary minerals that form on the surface of the slag grains control the leaching process at a material’s natural pH, since the leaching is governed by a surface phenomenon. The general SEM micrograph of the prehydrated slag grain with a reaction rim is shown in Figure 4. Portlandite and calcite are commonly present in weathered steel slags [46,47,48,49]. The occurrence of calcite could result in hydration and the carbonation of free lime [48], or the carbonation of calcium silicates [49]. In the Javornik slag heap it is more likely that the carbonation of calcium silicates is the predominant reaction mechanism, as calcite was frequently observed in the reaction rim that formed at the edge of the slag grain (Figure 4). The results of the SEM/EDS reveal that the reaction rims were composed of several different mineral phases, with calcium aluminate being the prevailing constituent. The formation of calcium aluminate hydrate phases (CAH) (e.g., ettringite, hydrocalumite) and magnesium aluminate hydrate (hydrotalcite) are attributed to the hydration reaction of the calcium aluminates (mayenite, tricalcium silicate, and brownmillerite). The reaction rim, composed of two CAH phases, is shown in Figure 5a. Microcrystalline hydrocalumite and CAH incorporating sulfate can be seen, and the typical microstructure of ettringite, described as “tiger stripe” morphology is evident [50]. The reaction rim of the slag grain, composed of calcite and ettringite, is shown in Figure 5b. Several authors have highlighted the high potential of ettringite to substitute SO_4_^2−^ with oxyanions such as CrO_4_^2−^ and MoO_4_^2−^ [51,52,53,54]. It has been suggested that the solid solution formation of ettringite (sulphate-oxyanion ettringite) is a controlling mechanism for the leaching of oxyanions [55], as in the case of Cr(VI) immobilization in a cement composite [56]. Zhang et al. (2003) [51] and Segni et al. (2006) [57] also reported the potential of hydrocalumite to (partially) substitute OH^−^ with anions of various sizes (CrO_4_^2−^, MoO_4_^2−^, and SeO_4_^2−^, V_2_O_7_^4−^), resulting in the immobilization of these potentially toxic elements. Further, mineral with excellent anion-exchange properties is assigned also to a hydrotalcite (Rybka et al., 2021) [58].

### 3.3. Groundwater (GW) Monitoring

**Figure 6:**
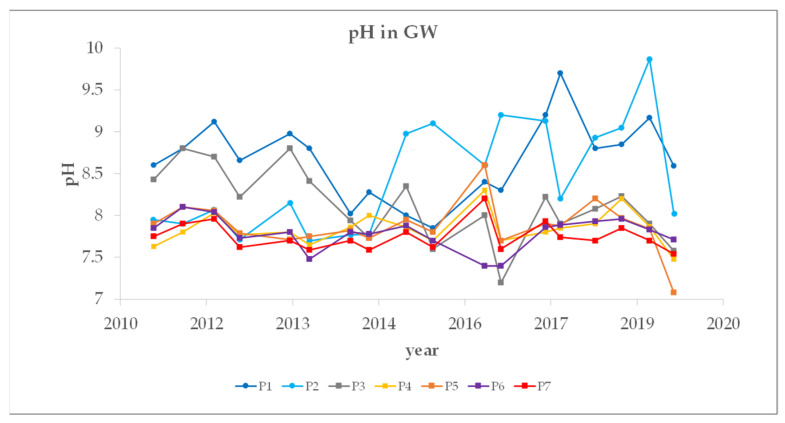
GW monitoring of pH over the period 2011–2019.

The GW monitoring continues to be in operation. The GW monitoring data are available from the period between 2011 and 2019. The samples were collected from seven different locations, with locations P1 and P2 being reference points. The pH of the GWs is generally mildly alkaline, ranging from 7.1 to 9.9, with an average of 8.1 (Figure 6). Interestingly, the pH values from the reference piezometers (P1–P2) were slightly higher (average pH of 8.6) than those from the piezometers P3–P7 (average pH of 7.9). It must be pointed out that the landfill samples (individual samples (L1–L11) and composite landfill sample PTO are alkaline, with a leachate pH (with water as the leachate medium, since this represents the material’s natural pH) ranging between 9.8 and 12.3 (average pH of 11.6). When studying the disposal of stabilized waste, Van der Sloot et al. (2007) [59] observed high pHs in the alkaline materials and a neutral to very mildly alkaline pH in the percolation and run-off water, and suggested that the layer of soil at the bottom of the waste compartment buffers the pH in the percolation water. In our case, however, the base of the landfill is barren gravel with sand, which is very permeable, the estimated permeability coefficient (k) is between 2.1 and 8.8 × 10^−3^ ms^−1^. The GW is presumably in constant contact with the meteorite water, which percolates through the landfill site, but on the other hand it is also fed from the backwaters. There is also a lot of rainfall in this area, with the nine-year precipitation average being around 1582 mm per year. Over the past nine years the average pH values were 8.6 and 8.3 at the reference piezometers P1 and P2, respectively. Slightly higher pHs were also observed at P3 (average pH of 8.3) in comparison to the other piezometers, where very similar pH values (average pH of 7.8) were observed over the nine-year period (Figure 6). P3 is located on the south-eastern edge of the landfill, where the impact of the landfill is more pronounced, whereas the other piezometers (P4–P7) are located in the wider area influenced by the landfill. The depth of the P7 piezometer, which is located at 5 m, compared to the 20 m depth of the others, had no noticeable influence on the average pH.

In terms of the concentrations of major cations in the GW, the order of abundance is Ca^2+^ > Mg^2+^ > Na^+^ > K^+^, while for the major anions it is HCO_3_^−^ >SO_4_^2−^ >Cl^−^ (Table 4). The high carbonate level of the GW (average range of 192–486 mg/L) could also lead to a lower pH of the infiltrating water. In general, there were no significant differences between the major cation and anion contents of the reference (P1, P2) and test (P3–P7) piezometers. On average, slightly lower concentrations of Ca (36.6 mg/L) and SO_4_^2−^ (31.8 mg/L) were observed at P3 (located in an area more strongly influenced by the landfill) compared to those measured at P4–P7 (located in the wider area of influence). A slightly higher average Ca content, ranging from 60.4 to 65.0 mg/L, was also observed in P4–P7 compared to the reference (P1–P2), whose average Ca content ranged between 23.1 and 41.6 mg/L. Compared to the reference piezometers, higher concentrations were also observed at P6 (for Na, SO_4_^2−^, HCO_3_^−^) and P7 (for K, Mg, and HCO_3_^−^). Minor variations were observed between the values obtained from the piezometers at a depth of 20 m (P3–P6) and the piezometer at a depth of 5 m (P7). The content of major cations and anions were below the maximum permissible limits defined by WHO in all the GW samples [60].

Values of dissolved organic carbon (DOC) were low, ranging from 0.33 to 4.4 mgL^−1^. The formation of DOC and metal complexes may affect the mobilization and speciation of metals [61]. It has been shown that DOC present in groundwater polluted by landfill leachate has the ability to form complexes with Cd, Ni, Zn, Cu and Pb [62,63]. The results of geochemical modelling (see Section 3.4, ‘Geochemical modelling’), did not, however, show metal–DOC complexation.

The presence and fate of steel additives such as Cr, Ni, and Mo in the dumped EAF and ladle slags are an important factor in GW contamination. The most relevant trace metals are presented in Table 5. The concentrations of elements of environmental concern (Cr, Ni, Pb, Zn and Mo) in the GW samples were low, with the majority (Cr, Ni, Pb) being below the detection limits (of 1, 1, and 0.5, μg/L, respectively). In general, there were no significant differences between the samples taken at the reference piezometers (P1–P2) and those taken at the others (P3–P7). Mo was observed in comparable concentrations at P3–P7 and P1–P2, ranging on average between 1.45 and 21.3 μg/L. A similar observation was found for Zn, with average concentrations at the reference piezometers (P1–P2) ranging from 2.4 to 6.27 μg/L, compared to a range of 2.09 to 9.42 μg/L at the other locations (P3–P6), with the exception of P7, where a higher average concentration of Zn (186 μg/L) was observed. The noticeable difference in the Zn concentration may be due to the differing depth of the piezometers (P7 at a depth of 5 m, compared to the others at a depth of 20 m). Such a trend was not, however, observed for the other metals of environmental concern (Cr, Ni, Pb), nor for the major cations and anions. This leads us to the conclusion that a hot spot of Zn-rich waste is likely located near the location of the P7 piezometer.

It must also be mentioned that the concentrations of metals of environmental concern (Cr, Ni, Pb, Zn and Mo) at P3 (located in an area more strongly influenced by the landfill), were lower in comparison to those at P4–P7 (located in the wider area influenced by the landfill). The concentration of Cr, Ni and Pb at P3 were even below the LOD (of 1, 1, and 0.5, μg/L, respectively).

Additionally, the GW data was compared with that from the study of Bayless (1998) [16] in order to roughly evaluate if the concentrations of the elements measured lie within similar ranges. This comparison must be taken with caution, however, because of differences in the chemical composition of the slags and other wastes disposed, differing aquifer compositions, and different sampling locations and techniques. In the study by Bayless (1998) [16], the GW samples were collected from Bairstow landfill in the US. The order of abundance of the major cations in GW from slag wells was Ca^2+^ > Na^+^ > K^+^ > Mg^2+^, with slightly higher concentrations of Ca (210–270 mg/L), Na (34–220 mg/L), and K (81–120 mg/L), and lower concentrations of Mg (27–41 mg/L) in comparison to the current study. Similar to as in our case, the concentrations of metals of environmental concern were low, with Cr ranging from 2 µg/L to 4 µg/L, Ni from 9 µg/L to 13 µg/L, Zn from 2 µg/L to 3 µg/L and Mo from 8 µg/L to 200 µg/L. In general, the metal concentrations observed in the study by Bayless (1998) [16] were higher than in our study, with the exception of Zn.

### 3.4. Geochemical Modelling

**Figure 7:**
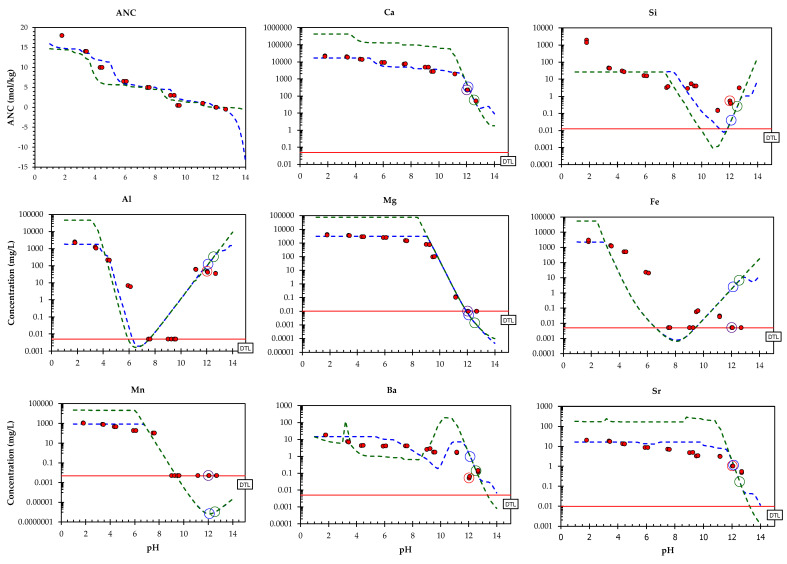
The measured and predicted leaching behaviour of major (Ca, Si, Mg, Al, Fe), some other elements (Mn, Sr, Ba) as a function of pH. Solid circles: the pH dependence test; lines: prediction at L/S = 10 mL/g (green), prediction at L/S = 0.3 mL/g (blue), LOD (limit of detection; solid red line).

**Figure 8:**
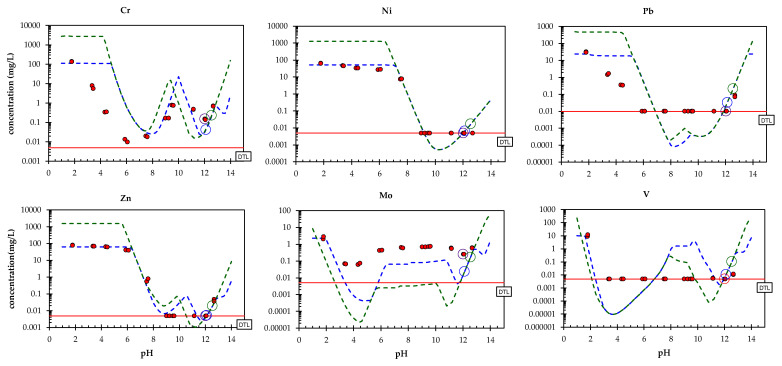
The measured and predicted leaching behaviour of elements of environmental concern (Cr, Ni, Pb, Zn, Mo, V) as a function of pH. Solid circles: the pH dependence test; lines: prediction at L/S = 10 mL/g (green), prediction at L/S = 0.3 mL/g (blue), LOD (limit of detection; solid red line).

Based on the results of preliminary modelling [34], a model run was performed with a slightly modified redox state, i.e., pH+pe = 14. The modelling was based on multi-element modelling, taking into account all elements, meaning it is difficult to simultaneously provide optimal descriptions for all of the elements (due to limitations in the accuracy, stability constants and measurements achievable). The resulting chemical speciation fingerprint can be found in the Supplementary Materials. Results of the model for the composite landfill sample PTO (only for selected elements) are given in Figure 7 and Figure 8, along with the original pH dependence data. For other elements refer to the Supplementary Materials. In all cases, the test data are given for comparison with the modelling results at both L/S = 10 mL/g and L/S = 0.3 mL/g (with all the other parameters remaining the same) in order to estimate the accuracy of the model by means of the selected mineral and sorption parameters for both a wide pH range as well as a wide L/S range [24,64,65]. In the case of many elements (Ca, Si, Al, Mg, Fe, Mn, Ni, Pb, Zn, V), the concentrations measured at L/S = 10 correspond well with simulations from the model. At neutral to middle acidic pH values the predicted values of Cr and Pb were overestimated, whereas in the same pH range Fe was underestimated. There is still room for improvement in the prediction of Mo, where the shape of the leaching curve is captured, but not to the correct level. The modelling of oxyanions (CrO_4_^2−^, MoO_4_^2−^, VO_4_^2−^) is challenging, however, since thermodynamic data for these trace oxyanions are not as abundant as for other trace metals, and sometimes even non-existent. Overall, however, the results of the modelling indicate that the solubility control of the solution concentrations, as described by the assemblage of minerals and sorptive phases, provides a reasonable description of the EAF/ladle slag heap over a wide pH range under mildly reducing conditions, as are the conditions for the laboratory-handled landfill samples.

As previously described, the leaching curves are the product of complex chemical processes in both the leachate solution and the solid phase of the waste material [24,64,65]. The mineral phases presumed to control the leaching from the composite landfill sample PTO (K_sp_ = solubility products) are presented in the Supplementary Materials.

**Figure 9:**
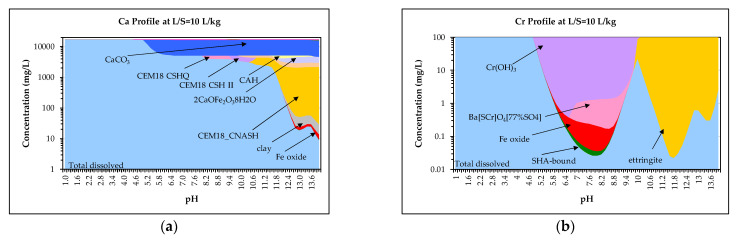
Partitioning of (**a**) Ca and (**b**) Cr across different chemical phases, as obtained from the modelling.

The partitioning of Ca and Cr in the liquid and solid phases is highlighted in Figure 9 (presented as a function of pH). For all other elements refer to the Supplementary Materials. Figure 9 illustrates that different processes control the leaching of Ca and Cr at different pH values.

The leaching of Ca seems to be adequately described by calcite (CaCO_3_), and to a lesser extent by hydrates of the calcium ferrites (2CaO∙Fe_2_O_3_∙10H_2_O), ettringite and the solid solution of CEM18_CNASH. CEM18_CNASH represents calcium (alkali) aluminosilicate hydrate (C-(N-)A-S-H) [66]. Calcite has frequently been suggested as a solubility-controlling phase for the leaching of Ca from weathered slags at a material’s natural pH [46,67]. As identified in the studies by Huijgen et al. (2006) [67] and Van Zomeren et al. (2001) [46], and also confirmed in our study (see Section 3.2, ‘Mineralogical characterization’), calcite formation primarily occurs on the grains’ reaction rims. Hence, rather than through solid-state conversion, the carbonation reaction appears to take place in two subsequent steps; first, calcium is leached from the Ca-minerals, and then subsequently it reacts with the dissolved carbonate to form calcite, which precipitates on the surface of the steel slag particles. With respect to the other major elements, leaching at a material’s natural pH seems to be adequately described by calcium (alkali) aluminosilicate hydrate (CEM18_CNASH) for Si, by brucite for Mg, and by CAH (calcium aluminate hydrates) for Al.

The leaching of Cr at materials’ natural pH was most likely controlled by ettringite solid solution (Figure 9). Several authors have pointed out the possibility of SO_4_^2^^−^ being substituted by CrO_4_^2^^−^ [51,52,53,54]. Ettringite was also experimentally identified in the PTO sample investigated (see Section 3.2, ‘Mineralogical characterization’). The geochemical models showed that the leaching of Ni could be described by Ni(OH)_2_, Pb by Pb(OH)_2_, and to a lesser extent by sorption onto hydrated particles of iron oxide, Zn by Ca(OH)_2_·Zn(OH)_2_, and Mo by ettringite. Additionally, the leaching of V could be described by sorption onto hydrated iron oxide particles, and Ba and Sr by co-precipitation in CaCO_3_.

The results from the geochemical modelling agree well with the previous study on fresh ladle slags, which investigated the leachability of freshly produced ladle slag derived from the production of austenitic and ferritic stainless steel and electrical and structural steel [24]. Please refer to the Supplementary Materials. The results of both models showed that the leaching behaviour of the fresh and composite landfill sample parameters investigated was, to a large extent, controlled by similar mineral and sorptive phases. As was already assumed in the previous study, however, weathering had an effect on the mineralogy of the disposed steelmaking slags. Calcite, portlandite, brucite, ettringite, hydrocalumite and hydrotalcite were assigned as secondary mineral phases, which to some extent control the leaching of the parameters investigated at the materials’ natural pH. The modelling also confirms that mechanisms such as co-precipitation with calcite also play an important role in leaching, as was suggested in the case of Ba and Sr. In the study by Loncnar et al. (2016) [24], however, the concentrations of Pb and Zn at the materials’ natural pH were below the LOD, which indicates that waste material other than EAF slag and ladle slag have probably also been landfilled at the Javornik slag heap.

## 4. Discussion

**Figure 10:**
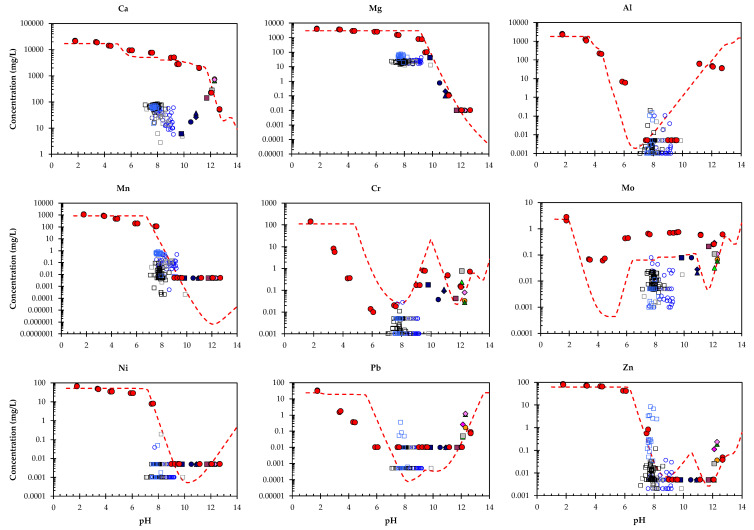
Comparison between the predicted leaching behaviour (red line) and the measured pH dependent leaching of the PTO sample (marked by red dots), the GW monitoring (open blue circles = reference piezometers (P1–P2); open grey squares = P3; open black squares = P4–P6; open blue square = P7) and the individual samples from the EAF and ladle slag heap (solid symbols; L3, L4, L7, L11 = darker blue symbols; others (L1, L2, L5, L6, L8, L9, L10) = various symbols; batch at L/S = 10 mL/g).

The results of the batch tests of the individual samples (L1–L11) and the GW monitoring data (P1–P7) are shown in Figure 10, placed in context against the pH dependent leaching data of the composite landfill sample PTO. For all other elements refer to the Supplementary Materials.

As can be seen from Figure 10, there was no big difference between the concentrations measured at the reference piezometers (P1–P2) (marked by open blue circles) and those measured at the other piezometers in the landfill area (P3—open grey square; (P4–P6)—open black square; P7—open blue square), indicating that the landfill had a limited influence on the quality of the GW. The leachate dilution factor was estimated based on an estimated flow rate of 20.7 m/day, as was reported for 2019. From the daily flow rate an average yearly flow rate was calculated (7.556 m/year), which, based on an assumed GW depth of 5 m, equates to 37.780 m^3^ of water per m^2^ of landfill. Taking into account that the average infiltration (2011–2019) in the Jesenice area is 1582 mm/yr, which translates to 1.58 m^3^/yr for 1 m^2^ of landfill. Based on assumed 500 m of landfill length in the flow direction, considering average infiltration 1.58 m^3^/yr for 1 m^2^ of landfill, which translates to 790 m^3^ of water/year. Diluted factor is then calculated as 37.780 m^3^ of water per m^2^ divided by 790 m^3^. This means that the leachate is diluted by approx. 48 times and it is not expected that elements of environmental concern would be released from the landfill into the GW.

The main difference between the results of the batch tests and those based on the pH dependence tests, on the one hand, and the GW data, on the other, is contact with the landfill material, which is absent in the case of the GW monitoring. For the majority of elements (Ca, Mg, Cr, Mn, Mo, Ni) the GW data shows lower concentrations. In the case of Zn, however, the GW data aligns, to some extent, with the pH dependent curve of the composite landfill sample PTO. Furthermore, no noticeable differences were observed between the measurements taken by the piezometers located at a depth of 20 m (P1–P6) and the piezometer located 5 m deep, with the exception of the Zn values, for which the data falls close to the pH dependent curve of the composite landfill sample PTO in the case of P7 (Figure 10). The GW data for the other parameters analysed at P7 did not, however, show such a trend. Presumably a hot spot of Zn-rich waste is located near the location of the P7 piezometer.

**Figure 11:**
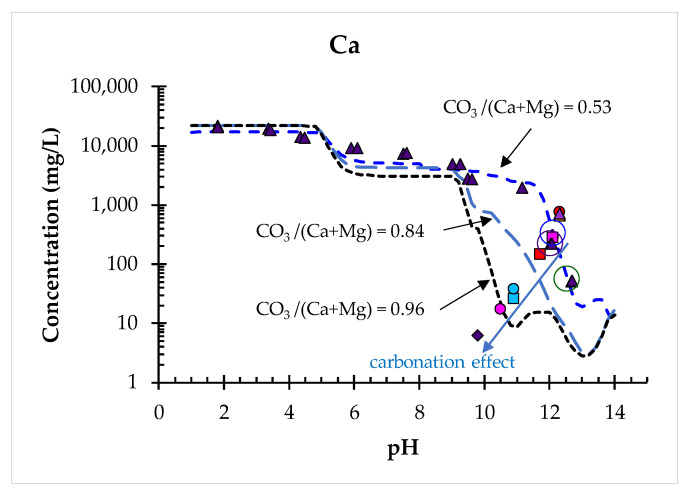
Comparison of the pH dependent leaching in the composite landfill sample PTO (marked by purple triangles) and the individual samples from the EAF/ladle slag heap (L3 = pink circle, L4 = dark purple diamond, L7 = blue circle and L11 = blue square, others (L1, L2, L5, L6, L8, L9, L10) = various symbols; batch at L/S = 10 mL/g) with data from modelling at different CO_3_/(Ca+Mg) molar ratios.

In the case of the majority of the elements investigated, the results of the batch test plot align with the pH dependent leaching curve for the composite landfill sample PTO, indicating that the same phases that control solubility control the leachability at the pH observed (Figure 10). In the case of Ca, slightly lower concentrations were measured in some individual samples (L3, L4, L7 and L11). At the same time a lower pH was observed in these samples. Both indicate that the carbonation of these spot samples was more pronounced than in others. This is in line with the findings of Huijgen et al. (2005) [49], who observed that the native pH of steel slag samples decreases with an increasing degree of carbonation. Additional modelling with different CO_3_/(Ca+Mg) molar ratios was used to show the response at different levels of carbonation. As can be seen from Figure 11, the individual samples L3, L4, L7 and L11 show different degrees of carbonation. Model runs were carried out with CO_3_/(Ca+Mg) molar ratios of 0.53 (composite landfill sample, as tested), 0.84 and 0.96. The model captures the different degrees of carbonation. In comparison to fresh slag the carbonate level in the composite landfill sample PTO is already quite high (molar ratio CO_3_/(Ca+Mg) = 0.53). It seems that the alkaline steel slag and the unsaturated conditions in the slag landfill have a pronounced effect on the carbonation of the deposited material. The modelling results indicate that the slag acts as a CO_2_ suction pump. As can be seen in Figure 11, the composite landfill sample PTO reached partial saturation, whereas at the individual sampling locations (L3, L4, L7 and L11) the carbonation was already far higher. Since the samples showed more pronounced carbonation at both sampling depths (0.2 m and 6 m), it is assumed that historically slag was not deposited evenly across the landfill in layers, but rather in individual piles within the landfill. These piles would have been exposed to atmospheric conditions for a long time before new slag was deposited on top of them, and thus more pronounced carbonation has occurred in these areas.

For the majority of the other parameters investigated, especially for the elements of environmental concern (Cr, Ni, Pb, Zn and Mo), the results of the batch test plot (L3, L4, L7 and L11) align with the pH dependent leaching curve for the composite landfill sample PTO (Figure 10), indicating that the same phases that control solubility also control leachability, despite the more pronounced carbonation in these samples. In the case of Cr, however, the pH of the individual samples (L3, L4, L7 and L11) falls between 10 and 11, with concentrations below the modelled pH dependent leaching curve, showing that one solubility phase is missing in this range, and that the leaching of Cr in this pH range is lower than was predicted by modelling.

## 5. Conclusions

This study used characterization leaching tests and geochemical speciation modelling to assess the long-term environmental impact of a steel slag heap (Javornik, Slovenia) containing ladle slags and EAF slag resulting from the production of carbon and stainless steel.

Detailed mineralogical characterization by XRF and SEM/EDS confirms the presence of spinel, dicalcium silicate (larnite, γ dicalcium silicate), calcium aluminates (mayenite, tricalcium aluminate), periclase and brownmillerite, all of which are minerals typically found in steel slags. Additionally, secondary mineral phases such as calcite, portlandite, brucite, ettringite, hydrocalumite and hydrotalcite were also found. Through geochemical modelling these latter phases were identified as solubility-controlling phases. Since being landfilled, the deposited slag has undergone an intensive carbonation process. At an elevated carbonate level (molar ratio CO_3_/(Ca+Mg = 0.53), geochemical modelling appropriately describes the leaching behaviour of the parameters observed (major and elements of environmental concern: Cr, Ni, Pb, Zn, Mo). The carbonation was even more pronounced in certain areas, showing an even higher level of carbonate (CO_3_/(Ca+Mg = 0.96), meaning some parts of the landfill were almost fully carbonized. The carbonation had a limited effect on the leaching behaviour of the elements observed, however, particularly on the elements of environmental concern. Typically, Ca, Ba and Sr are affected, due to the formation of carbonate phases. For the other elements observed the chemical speciation fingerprint (CSF), developed from pH dependent leaching data from a composite of individual samples collected from different locations and depths, provides a good description of the release measured and the release-controlling phases on-site, which allows the release to be predicted under changing conditions of exposure such as in the case of carbonation. It seems that alkaline EAF and ladle slags act as a CO_2_ suction pump. Further research is, however, suggested in order to confirm this observation.

Overall, geochemical modelling showed that the leaching behaviour of the major elements and elements of environmental concern from the slag heap at the material’s native pH was primarily controlled by a limited set of mineral and sorptive phases. Based on a comparison of the individual slag samples with more sophisticated pH dependent data it can be concluded that the same minerals and sorptive phases control the leaching behaviour of elements of potential concern throughout the landfill, despite variations in elemental compositions of individual slag samples. It was also observed that leaching from the slag heap has a limited effect on the quality of the GW. In the GW samples with a higher than average pH (of around pH 7.9), low concentrations of the elements of environmental concern (Cr, Ni, Pb, Zn and Mo) were observed, with concentrations of most of the elements (Cr, Ni and Pb) being even below the detection limits (LOD_Cr,Ni_ = 1 µ/L and LOD_Pb_ = 0.5 µ/L, respectively). Furthermore, the data also showed no noticeable differences between the GW concentrations upstream and downstream of the landfill. A site-specific phenomenon was observed, in that a rapid flow of groundwater fed from the nearby Sava River was observed in the area of the landfill, meaning that the leachates from the slag heap were strongly diluted. Based on the consistent leaching behaviour of fresh EAF and ladle slag, as confirmed in earlier work [24,25], and the leaching behaviour of the composite landfill sample from Javornik, the beneficial use of fresh slag, and possibly even slag mining, could be an appropriate management practice. This topic requires further evaluation with regard to its potential applications.

The sampling and testing approach applied in this study, used to investigate the Javornik EAF/ladle slag heap, confirms earlier observations comparing laboratory and field studies [15]. Furthermore, in our case, the spatially distributed samples fit the pH dependent behaviour of the composite observed, so can thus either be represented by the same mineral and sorptive phases controlling the process, or can be explained by external influences such as carbonation. Placing individual location and depth samples in context with the data and modelling the results of the composite is an appropriate and cost-effective approach towards assessing the long-term environmental impact of an old metallurgical heap on the area’s soil and GW.

## Figures and Tables

**Table 1:** Sample IDs of the 11 individual samples and 1 mixed sample sampled from the EAF/ladle slag heap in Javornik.

Individual Sample	L1	L2	L3	L4	L5	L6
Sample depth	200 mm	200 mm	200 mm	200 mm	200 mm	200 mm
Individual sample	L7	L8	L9	L10	L11	
Sample depth	6000 mm	6000 m	6000 mm	6000 mm	6000 mm	
Composite sample	PTO	mix (L2-L11)				

**Table 2:** Chemical composition of the composite landfill sample (wt.%) in comparison with the range of chemical compositions reported in the literature. EAF slag and ladle slags data are adopted from Yildirim et al. (2011) [35].

	CaO	SiO_2_	Al_2_O_3_	MgO	FeO	Cr_2_O_3_	MnO
PTO	26.7 ± 0.3	14.6 ± 0.2	7.0 ± 0.1	8.5 ± 0.1	20.6 * ± 1	2.2 ± 0.3	3.7 ± 0.1
EAF slag	24.5–60	9–20	2–12.2	5–15	5.6–34.4	-	2.5–8
ladle slag	30–60	2–35	5–35	1–12.6	0–15	-	0.5–5

* Total Fe expressed as FeO.

**Table 3:** Variation in the chemical composition of the landfill samples (wt.%).

Samples	CaO	SiO_2_	Al_2_O_3_	MgO	Fe_tot_	Cr_2_O_3_	MnO	S	C
L1–L6	35 ± 5	14 ± 2	10 ± 3	13 ± 3	13 ± 3	2.9 ± 0.5	2.6 ± 0.9	0.5 ± 0.2	2.7 ± 0.6
L7–L11	23 ± 4	20 ± 6	8 ± 3	9 ± 4	14 ± 2	1.5 ± 0.8	3.5 ± 1.9	0.28 ± 0.06	1.6 ± 0.4

**Table 4:** Range (min and max values) and average concentrations of anions and major cations (mg/L) in the GW over the period 2011–2019 (*n* = 126). The reference piezometers designated P1 and P2, respectively.

	Ca	K	Mg	Na	SO_4_^2−^	Cl^–^	HCO_3_^−^
	Min–Max Average	Min–Max Average	Min–Max Average	Min–Max Average	Min–Max Average	Min–Max Average	Min–Max Average
P1	5.70–69.7 23 ± 18	2.50–6.36 3.6 ± 1.2	16.0–42.3 26 ± 5	1.70–26.3 11 ± 4	17.4–125 50 ± 36	17.1–28.3 21 ± 3	12 –363 192 ± 56
P2	4.90–77.9 42 ± 23	1.50–11.5 5 ± 3	13.0–29.1 20 ± 5	9.50–20.3 14 ± 3	1.72–78.6 41 ± 23	14.9–24.9 19 ± 3	101–313 194 ± 56
P3	2.80–71.3 37 ± 17	1.30–6.10 2.4 ± 1.3	18.5–26.3 22 ± 2	8.70–15.1 12 ± 2	5.19–74.2 32 ± 17	13.8–25.0 18 ± 3	138 –296 202 ± 47
P4	16.7–84.9 69 ± 17	2.70–8.04 5 ± 2	17.3–30.2 25 ± 3	1.70–16.4 12 ± 4	14.0–130 82 ± 29	13.2–25.2 19 ± 3	147–311 229 ± 47
P5	9.66–49.2 65 ± 10	1.40–4.88 3 ± 1	14.0–26.4 20 ± 3	5.10–15.4 11 ± 3	8.00–73.5 45 ± 18	7.54–23.8 15 ± 5	149–301 219 ± 44
P6	61.7–81.4 71 ± 5	4.60–11.5 6.8 ± 1.6	19.8–24.1 22 ± 2	7.30–18.9 15 ± 3	9.00–95.8 64 ± 22	15.8–29.3 21 ± 3	134–2.340 359 ± 505
P7	14.7–74.3 60 ± 13	1.35–6.10 8.13 ± 1.4	19.5–77.1 54 ± 1	1.42–15.5 8.4 ± 3.6	4.50–28.8 14 ± 8	4.05–14.3 7.7 ± 3.3	181–1.151 486 ± 194

**Table 5:** Range and average metal concentrations (µg/L) in the GW over the period 2011–2019 (*n* = 126). The reference piezometers designated P1 and P2, respectively. ‘<’ denotes the value was below the given limit of detection (LOD). Where measurements were below the LOD, the value equal to ½ LOD was used for calculating the average concentration.

	Cr	Ni	Pb	Zn	Mo
	Min–Max Average	Min–Max Average	Min–Max Average	Min–Max Average	Min–Max Average
P1	<1 <1	<1 <1	<0.5 <0.50	<2– 36 6.27 ± 10.3	<1–6.7 1.53 ± 1.52
P2	<1–27.4 3.3 ± 8.1	<1 * <1	<0.5 <0.5	<2–10.3 2.4 ± 2.6	<1–78.9 21.3 ± 19.2
P3	<1 <1	<1 * <1	<0.5 <0.5	<2–5.7 2.09 ± 2.1	<1–17.4 0.83 ± 0.57
P4	<1–10.9 1.9 ± 2.5	<1–1.1 0.53 ± 0.32	<0.5 <0.5	<2–23.6 8.9 ± 7.1	<1–11 5.87 ± 3.34
P5	<1–1.5 0.74 ± 0.37	<1 <1	<0.5 <0.5	<2–33.1 9.42	<1–14 6.34 ± 3.89
P6	<1–5.1 1.15 ± 1.14	<1 <1	<0.5 <0.5	<2–29.9 9.35 ± 8.0	13.0–23.7 18.5 ± 3.62
P7	<1 <1	<1–1.8 * 0.58 ± 0.32	<0.5–0.6 ** 0.28 ± 0.09	34.5–308 *** 186 ± 100	<1–11.1 1.45 ± 2.47

* Ni: 3 outliers excluded (up to 100 times higher conc.: P2, P3, and P7), ** Pb: 4 outliers excluded (up to 250 times higher conc.: P7), *** Zn: 6 outliers excluded (up to 250 times higher conc.: P6, and P7).

## Data Availability

Data is contained within the article or supplementary material.

## Supplementary Materials

The following are available online at https://www.mdpi.com/article/10.3390/toxics10010010/s1.
