# Supplementary material for: Leaching and Geochemical Modelling of an Electric Arc Furnace (EAF) and Ladle Slag Heap
Source: Toxics. 2022 Jan 1;10(1):10. doi: 10.3390/toxics10010010 (PMC8778747; doi:10.3390/toxics10010010)

toxics MDPI

Supplementary Material

Leaching and geochemical modelling of an electric arc furnace (EAF) and ladle slag heap

Mojca Loncnar[1*], Ana Mladenovič[2], Vesna Zalar Serjun[2], Marija Zupančič[3], Hans A. van der Sloot[4]

Table S1: Range and average concentration of additional parameters (µg/L) in GW during period 2011 – 2019 (n =126). The reference piezometers designated P1 and P2, respectively. '<' denotes the value was below the given limit of detection (LOD). Where measurements were below the LOD, the value equal to ½ LOD was used for calculating the average concentration.

	**Al**<br>**min – max**<br>**average**	**B**<br>**min – max**<br>**average**	**Ba**<br>**min – max**<br>**average**	**Fe**<br>**min – max**<br>**average**	**Mn**<br>**min – max**<br>**average**	**TOC**<br>**min – max**<br>**average**	**Cl⁻**<br>**min – max**<br>**average**
P1	<1–39*<br>4.1±9.9	25–198**<br>108±32	<5–108***<br>17±25	<10–23<br>6.7±4.6	27.6–358*****<br>99±78	470–3,940<br>1,600±900	17,100–28,300<br>21,000±2,800
P2	<1–195*<br>1.1±1.3	24.2–123<br>58±33	<5–58.5***<br>31±16	<10–13.5****<br>5.7±2	<3–62.1*****<br>21±22	150–3,640#<br>1,700±1100	14,900–24,900<br>19,000±2,800
P3	<1–19.5*<br>1.8±4.6	11–95<br>39±24	<5–62<br>27±18	<10<br>5.2±0.9	<3–354*****<br>107±77	330–4,300<br>1,400±1100	13,800–25,000<br>17,900±3,200
P4	<1–31.7<br>3.4±7.6	57–175<br>105±35	30.7–86.8<br>46±15	<10<br><10	<3–38.4<br>14±11	150–3,510<br>1,500±1000	13,200–25,200<br>18,500±3,300
P5	<1–2.7*<br>1.04±0.76	15.1–70<br>42±13	28.8–74<br>43.6	<10<br><10	<3–34.8*****<br>6.6±7.6	150–4,400<br>1,400±1300	7,540–23,800<br>14,700±4,900
P6	<1–4.0<br>1.12±0.95	30–108<br>68±20	37.4–93.7<br>54±15	<10<br><10	<3–27.3*****<br>5.1±6.5	518–3,240<br>1,400±800	15,800–29,300<br>20,900±3,000
P7	<1–3.2*<br>1.03±0.87	22.7–495<br>279±159	20.2–1034<br>674±224	<10–32.7<br>7.7±7.4	7.2–853<br>563±168	570–4,170<br>2,100±900	4,050–14,300<br>7,700±3,300

* Al: 7 outliers excluded (P1, P2, P3, P5, P7), ** B: 1 outlier excluded (P1), *** Ba: 2 outliers s excluded (P1, P2), ****Fe: 1 outlier excluded (P1), *****Mn: 5 outliers excluded (P1, P2, P3, P5, P6),#TOC 1 outlier excluded (P2)

Table S2: Chemical speciation fingerprint

**Model Parameters**	
Clay (mg/kg)	8.000E+04
Hydrous Ferric Oxide (mg/kg)	800.0
L/S (L/kg)	10.00
pE	1.580
pH	12.42
Solid Humic Acid (mg/kg)	14.87

Reactant concentration

**Reactant**	**mg/kg**		**mg/kg**		**mg/kg**		**mg/kg**
Ag	1.079E-07	CO32-	1.700E+05	Li	17.27	Sb	5.798
Al	1.834E+04	Cr	1118	Mg	3.048E+04	Se	0.05013
As	5.026	Cu	153.1	Mn	8.362	Sn	4.474
Ba	149.4	F	4254	Mo	23.28	S	1138
Br	7.990E-08	Fe	2.397E+04	Na	1147	Sr	163.7
Ca	1.703E+05	B	168.2	Ni	521.5	Th	2.320E-07
Cd	1.941	Si	1.142E+04	NO3	30.00	U	2.380E-07
Cl	42.90	Hg	2.006E-07	Pb	237.9	V	99.39
Co	6.742	K	207.1	P	988.7	Zn	629.9

Figure S1: The measured and predicted leaching behaviour of additional elements as a function of pH. Triangle: the pH dependence test; lines: prediction at L/S = 10 mL/g (green), prediction at L/S = 0.3 mL/g (blue), LOD (limit of detection; solid red line)

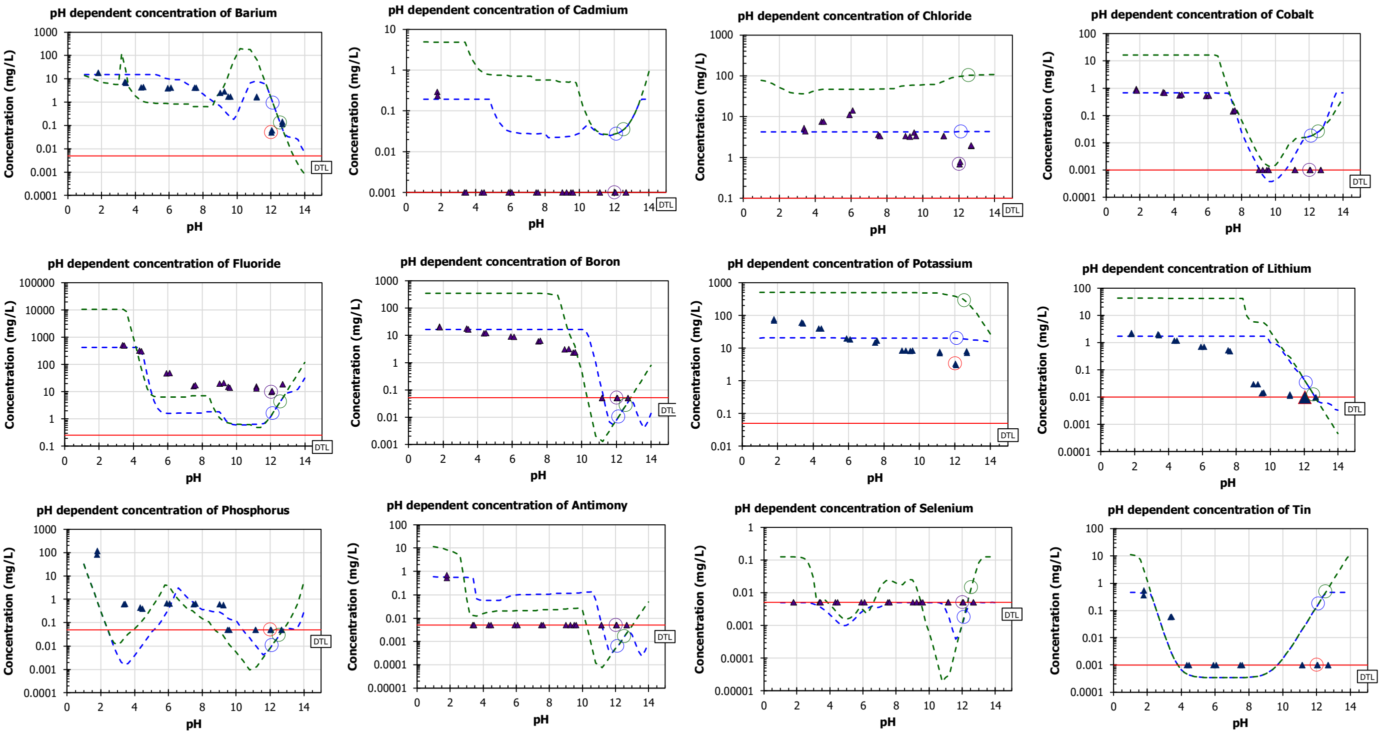

Table S3: The mineral phases

Solid Solutions

Name	End Member	Log(K)	Reaction
CEM18_CNASH_ss	CEM18_5CA	7.002	CEM18_5CA + 0.25 H+ -> 0.25 Al[OH]4- + 1 CEM18_CNASH_ss + 1.25 Ca+2 + 0.25 H2O + 1 H2SiO4-2
	CEM18_T2C_CNASHss	-2.720	CEM18_T2C_CNASHss + 1 H+ -> 1 CEM18_CNASH_ss + 1.5 Ca+2 + 2 H2O + 1 H2SiO4-2
	CEM18_T5C_CNASHss	10.34	CEM18_T5C_CNASHss -> 1.25 CEM18_CNASH_ss + 1.25 Ca+2 + 1.25 H2O + 1.25 H2SiO4-2
	CEM18_TobH_CNASHss	21.90	CEM18_TobH_CNASHss -> 1.5 CEM18_CNASH_ss + 1 Ca+2 + 1 H+ + 0.5 H2O + 1.5 H2SiO4-2
CEM18_CSH_II_ss	CEM18_Jennite	-6.533	CEM18_Jennite + 1.333334 H+ -> 1 CEM18_CSH_II_ss + 1.666667 Ca+2 + 1.766667 H2O + 1 H2SiO4-2
	CEM18_Tob_II	11.95	CEM18_Tob_II -> 1 CEM18_CSH_II_ss + 0.833333 Ca+2 + 0.333334 H+ + 0.166666 H2O + 1 H2SiO4-2
CEM18_CSHQ_ss	CEM18_CSHQ_JenD	-13.70	CEM18_CSHQ_JenD + 1.6666 H+ -> 0.6667 CEM18_CSHQ_ss + 1.5 Ca+2 + 2.6666 H2O + 0.6667 H2SiO4-2
	CEM18_CSHQ_JenH	0.7235	CEM18_CSHQ_JenH + 0.6666 H+ -> 1 CEM18_CSHQ_ss + 1.3333 Ca+2 + 1.5 H2O + 1 H2SiO4-2
	CEM18_CSHQ_TobD	1.633	CEM18_CSHQ_TobD + 0.33335 H+ -> 0.6667 CEM18_CSHQ_ss + 0.833375 Ca+2 + 1.3334 H2O + 0.6667 H2SiO4-2
	CEM18_CSHQ_TobH	14.85	CEM18_CSHQ_TobH -> 1 CEM18_CSHQ_ss + 0.6667 Ca+2 + 0.6666 H+ + 0.1667 H2O + 1 H2SiO4-2
ettr_ss	AsO4_Ettringite_ss	26.79	AsO4_Ettringite_ss + 1 H+ + 8 H2O -> 2 Al[OH]4- + 3 AsO4-3 + 6 Ca+2 + 1 ettr_ss
	Ba_Ettringite_ss	4.008	Ba_Ettringite_ss + 4 H+ + 8 H2O -> 2 Al[OH]4- + 6 Ba+2 + 3 SO4-2 + 1 ettr_ss
	BO3_Ettringite_ss	-46.87	BO3_Ettringite_ss + 7 H+ + 8 H2O -> 2 Al[OH]4- + 6 Ca+2 + 3 H2BO3- + 1 ettr_ss
	CrO4_Ettringite_ss	-8.592	CrO4_Ettringite_ss + 4 H+ + 8 H2O -> 2 Al[OH]4- + 6 Ca+2 + 3 CrO4-2 + 1 ettr_ss
	Ettringite_ss	-10.99	Ettringite_ss + 4 H+ + 8 H2O -> 2 Al[OH]4- + 6 Ca+2 + 3 SO4-2 + 1 ettr_ss
	Li-Ettringite_ss	-5.699	Li-Ettringite_ss + 4 H+ + 8 H2O -> 2 Al[OH]4- + 5 Ca+2 + 2 Li+ + 3 SO4-2 + 1 ettr_ss
	MoO4_Ettringite_ss	-9.592	MoO4_Ettringite_ss + 4 H+ + 8 H2O -> 2 Al[OH]4- + 6 Ca+2 + 3 MoO4-2 + 1 ettr_ss
	PO4_Ettringite_ss	39.10	PO4_Ettringite_ss + 1 H+ + 8 H2O -> 2 Al[OH]4- + 6 Ca+2 + 3 PO4-3 + 1 ettr_ss
	Sb[OH]6-_Ettringite_ss	-33.80	Sb[OH]6-_Ettringite_ss + 7 H+ + 17 H2O -> 2 Al[OH]4- + 6 Ca+2 + 3 Sb[OH]6- + 1 ettr_ss
	SeO4-2_Ettringite_ss	-8.592	SeO4-2_Ettringite_ss + 4 H+ + 8 H2O -> 2 Al[OH]4- + 6 Ca+2 + 3 SeO4-2 + 1 ettr_ss
	Sr_Ettringite_ss	4.008	Sr_Ettringite_ss + 4 H+ + 8 H2O -> 2 Al[OH]4- + 3 SO4-2 + 6 Sr+2 + 1 ettr_ss
	VO3_Ettringite_ss	-53.79	VO3_Ettringite_ss + 13 H+ + 2 H2O -> 2 Al[OH]4- + 6 Ca+2 + 3 VO2+ + 1 ettr_ss

Minerals

Name	Log(K)	Reaction
AA_2CaO_Fe2O3_8H2O[s]	-10.12	AA_2CaO_Fe2O3_8H2O[s] + 2 H+ -> 2 Ca+2 + 2 Fe[OH]4- + 5 H2O
AA_Brucite	-16.84	AA_Brucite + 2 H+ -> 2 H2O + 1 Mg+2
AA_Calcite	9.481	AA_Calcite -> 1 CO3-2 + 1 Ca+2
AA_CaO_Al2O3_10H2O[s]	7.490	AA_CaO_Al2O3_10H2O[s] -> 2 Al[OH]4- + 1 Ca+2 + 6 H2O
AA_Fe[OH]3[am]	16.60	AA_Fe[OH]3[am] + 1 H2O -> 1 Fe[OH]4- + 1 H+
AA_Magnesite	9.359	AA_Magnesite -> 1 CO3-2 + 1 Mg+2
AA_Portlandite	-22.80	AA_Portlandite + 2 H+ -> 1 Ca+2 + 2 H2O
Al4[OH]10SO4	69.30	Al4[OH]10SO4 + 6 H2O -> 4 Al[OH]4- + 6 H+ + 1 SO4-2
alpha-TCP	25.50	alpha-TCP -> 3 Ca+2 + 2 PO4-3
Antimocrandallite-exp	63.00	Antimocrandallite-exp + 8 H2O -> 3 Al[OH]4- + 1 Ca+2 + 3 H+ + 2 Sb[OH]6-
Austinite-therm	11.47	Austinite-therm + 1 H+ -> 1 AsO4-3 + 1 Ca+2 + 1 H2O + 1 Zn+2
Ba[SCr]O4[77%SO4]	10.13	Ba[SCr]O4[77%SO4] -> 1 Ba+2 + 0.23 CrO4-2 + 0.77 SO4-2
BaSrSO4[50%Ba]	8.221	BaSrSO4[50%Ba] -> 0.5 Ba+2 + 1 SO4-2 + 0.5 Sr+2
Ca[OH]2.Zn[OH]2_exp	-30.52	Ca[OH]2.Zn[OH]2_exp + 4 H+ -> 1 Ca+2 + 4 H2O + 1 Zn+2
Ca2V2O7	-8.750	Ca2V2O7 + 3 H+ -> 1 Ca+2 + 1.5 H2O + 1 VO2+
Ca3[BO3][PO4]	-2.216	Ca3[BO3][PO4] + 2 H+ -> 3 Ca+2 + 1 H2BO3- + 1 PO4-3
CaCO3_BaCO3	22.00	CaCO3_BaCO3 -> 1 Ba+2 + 2 CO3-2 + 1 Ca+2
CaCO3_Na2CO3	18.30	CaCO3_Na2CO3 -> 2 CO3-2 + 1 Ca+2 + 2 Na+
CaMoO4[c]	7.940	CaMoO4[c] -> 1 Ca+2 + 1 MoO4-2
Cd[OH]2[C]	-13.65	Cd[OH]2[C] + 2 H+ -> 1 Cd+2 + 2 H2O
Co2SiO4	6.289	Co2SiO4 + 2 H+ -> 2 Co+2 + 1 H2SiO4-2
Cr[OH]3[C]	66.68	Cr[OH]3[C] + 1 H2O -> 1 CrO4-2 + 5 H+ + 3 e-
Diaspore-exp	14.48	Diaspore-exp + 2 H2O -> 1 Al[OH]4- + 1 H+
FeVO4:2H2O_am	23.48	FeVO4:2H2O_am + 2 H2O -> 1 Fe[OH]4- + 1 VO2+
Fluorite	10.96	Fluorite -> 1 Ca+2 + 2 F-
LDH_Cd_zc	60.06	LDH_Cd_zc + 1 H2O -> 1 Al[OH]4- + 3 CO3-2 + 3 Cd+2 + 1 H+
LDH_Cu_zc	58.21	LDH_Cu_zc + 1 H2O -> 1 Al[OH]4- + 3 CO3-2 + 3 Cu+2 + 1 H+
Li2_CaO_Al2O3_SiO2_8H2O[s]	24.70	Li2_CaO_Al2O3_SiO2_8H2O[s] -> 2 Al[OH]4- + 1 Ca+2 + 3 H2O + 1 H2SiO4-2 + 2 Li+
Manganite	-25.27	Manganite + 3 H+ + 1 e- -> 2 H2O + 1 Mn+2
Ni[OH]2[s]	-10.80	Ni[OH]2[s] + 2 H+ -> 2 H2O + 1 Ni+2
OCP	46.90	OCP -> 4 Ca+2 + 1 H+ + 2.5 H2O + 3 PO4-3
PATCH_Ca5[OH][AsO4]3[c]	26.13	PATCH_Ca5[OH][AsO4]3[c] + 1 H+ -> 3 AsO4-3 + 5 Ca+2 + 1 H2O
Pb[OH]2[C]	-8.150	Pb[OH]2[C] + 2 H+ -> 2 H2O + 1 Pb+2
Pb2V2O7	0.9500	Pb2V2O7 + 3 H+ -> 1.5 H2O + 1 Pb+2 + 1 VO2+
Pb3[VO4]2	-3.070	Pb3[VO4]2 + 4 H+ -> 2 H2O + 1.5 Pb+2 + 1 VO2+
PbMoO4[cc]	13.36	PbMoO4[cc] -> 1 MoO4-2 + 1 Pb+2
SiO2[a]	24.64	SiO2[a] + 2 H2O -> 2 H+ + 1 H2SiO4-2
Sn[OH]2[s]	1.447	Sn[OH]2[s] + 2 H+ -> 2 H2O + 1 Sn+2
Strengite	48.00	Strengite + 2 H2O -> 1 Fe[OH]4- + 4 H+ + 1 PO4-3
Tenorite	-7.620	Tenorite + 2 H+ -> 1 Cu+2 + 1 H2O
Willemite	6.289	Willemite + 2 H+ -> 1 H2SiO4-2 + 2 Zn+2

Figure S2: Partiting of the elements of interest over different chemical phases as obtained from the modelling.

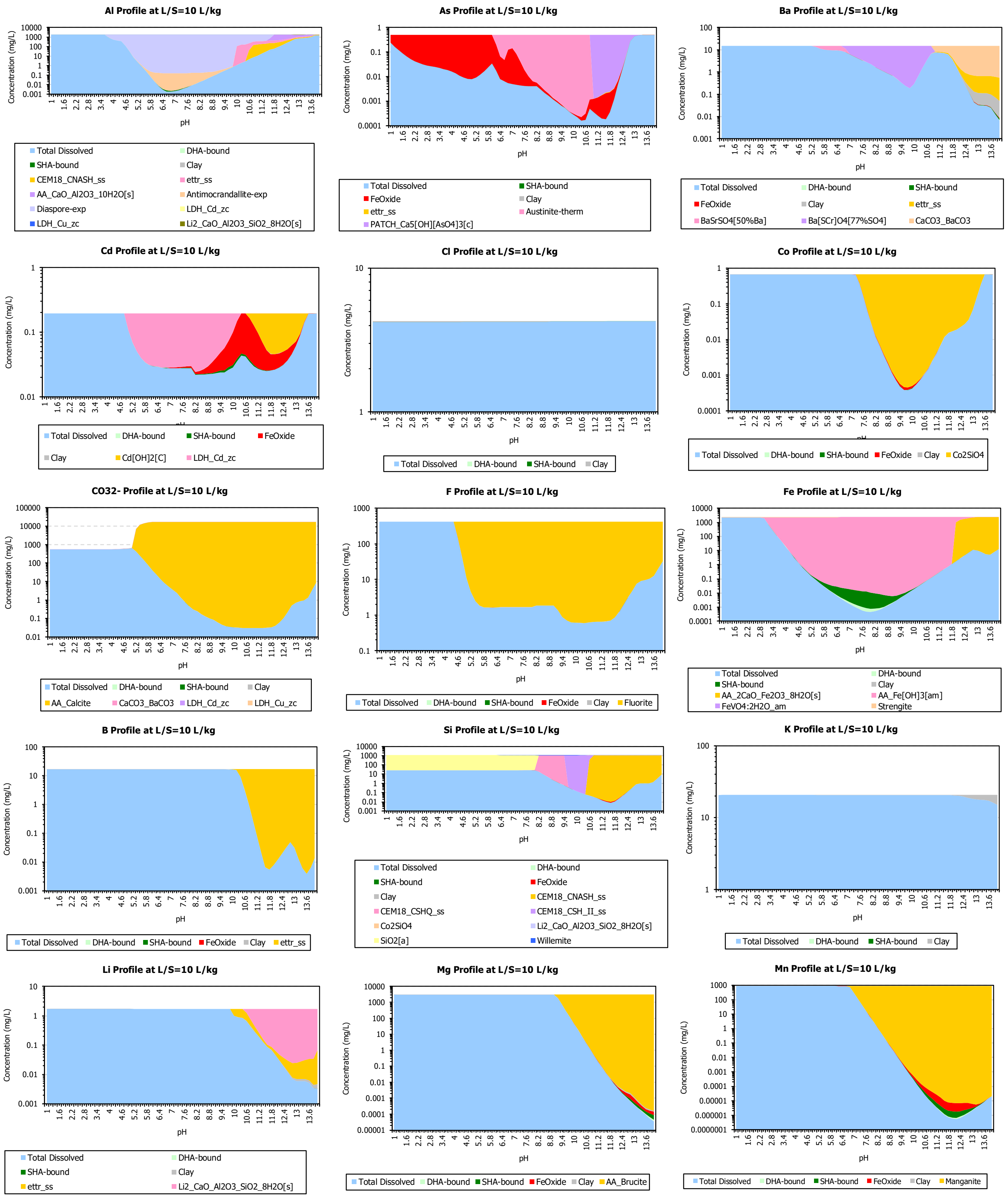

Figure S3: Partiting of the elements of interest over different chemical phases as obtained from the modelling

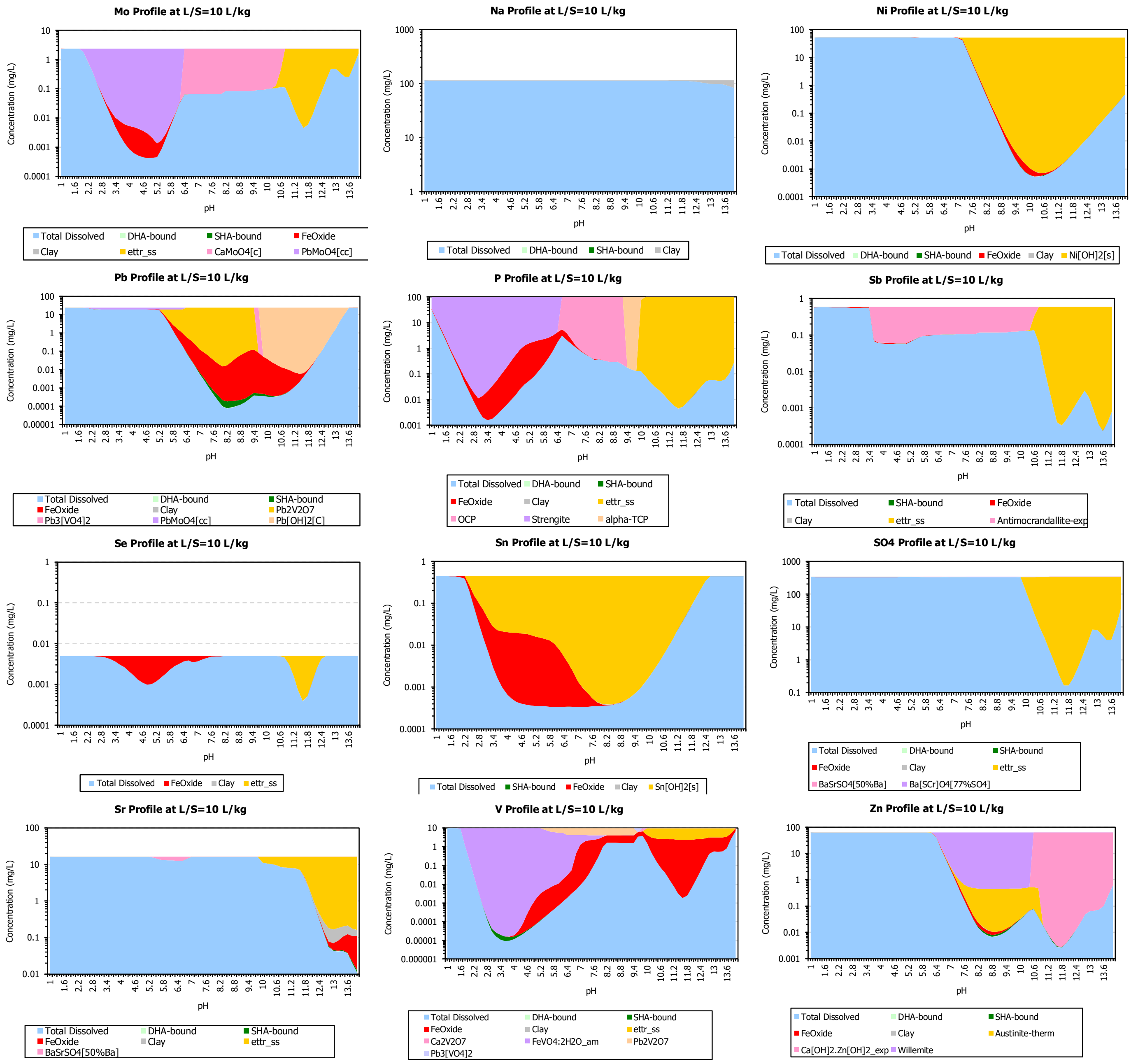

Figure S4: Comparison between the predicted leaching behaviour (red line) and the measured pH dependent leaching of the PTO sample (marked by red dots), the GW monitoring (open symbols) and the individual samples from the metallurgical heap (solid symbols; L3, L4, L7, L11 = darker blue symbols; others (L1, L2, L5, L6, L8, L9, L10) = various symbols) batch at L/S = 10 mL/g).

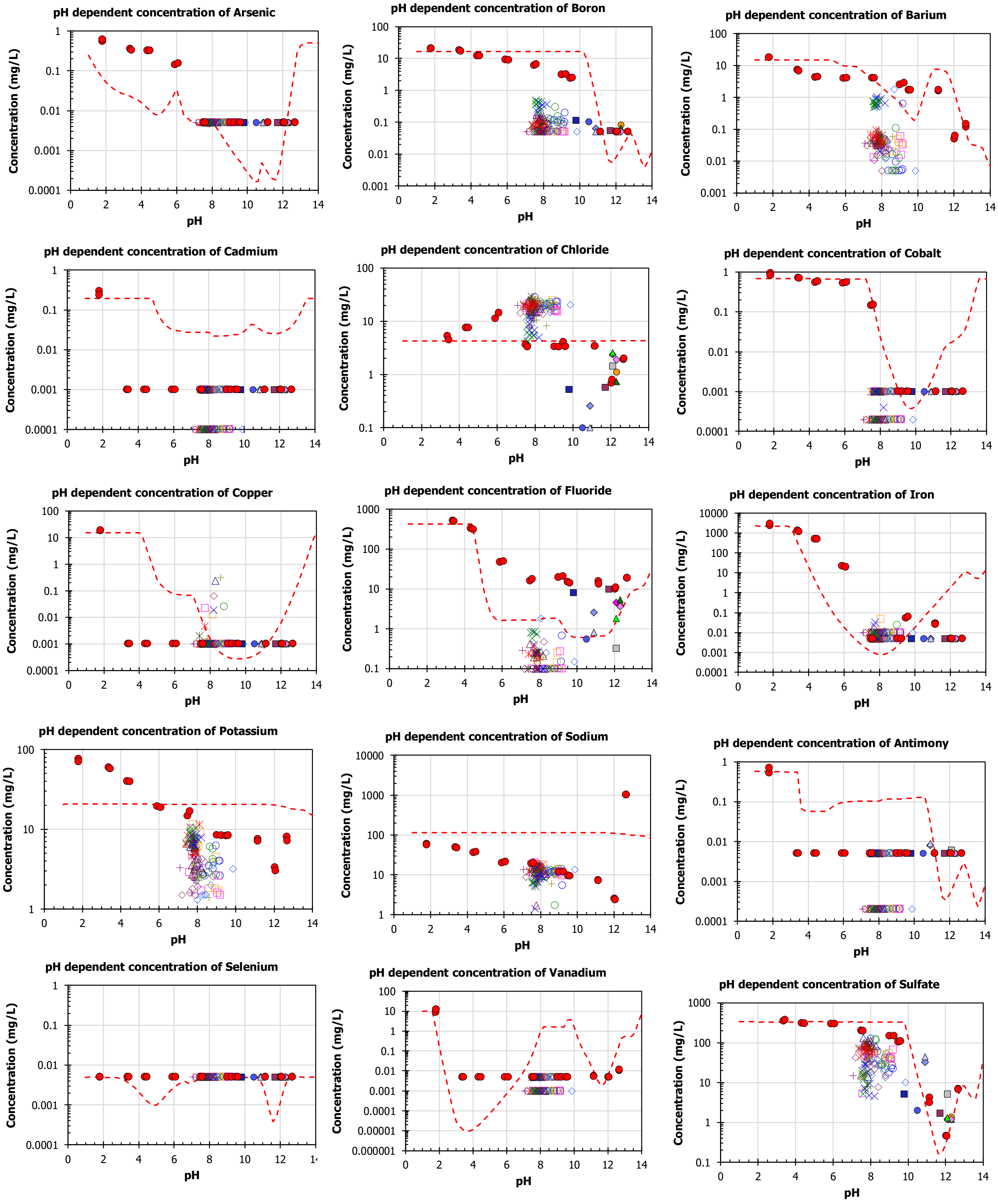

Figure S5: Comparison between the predicted leaching behaviour (red dashed line) and the measured pH dependent leaching of the composite landfill sample PTO (red circle), "fresh" EAF S slag (brown triangle) and "fresh" ladle slag (green circle).

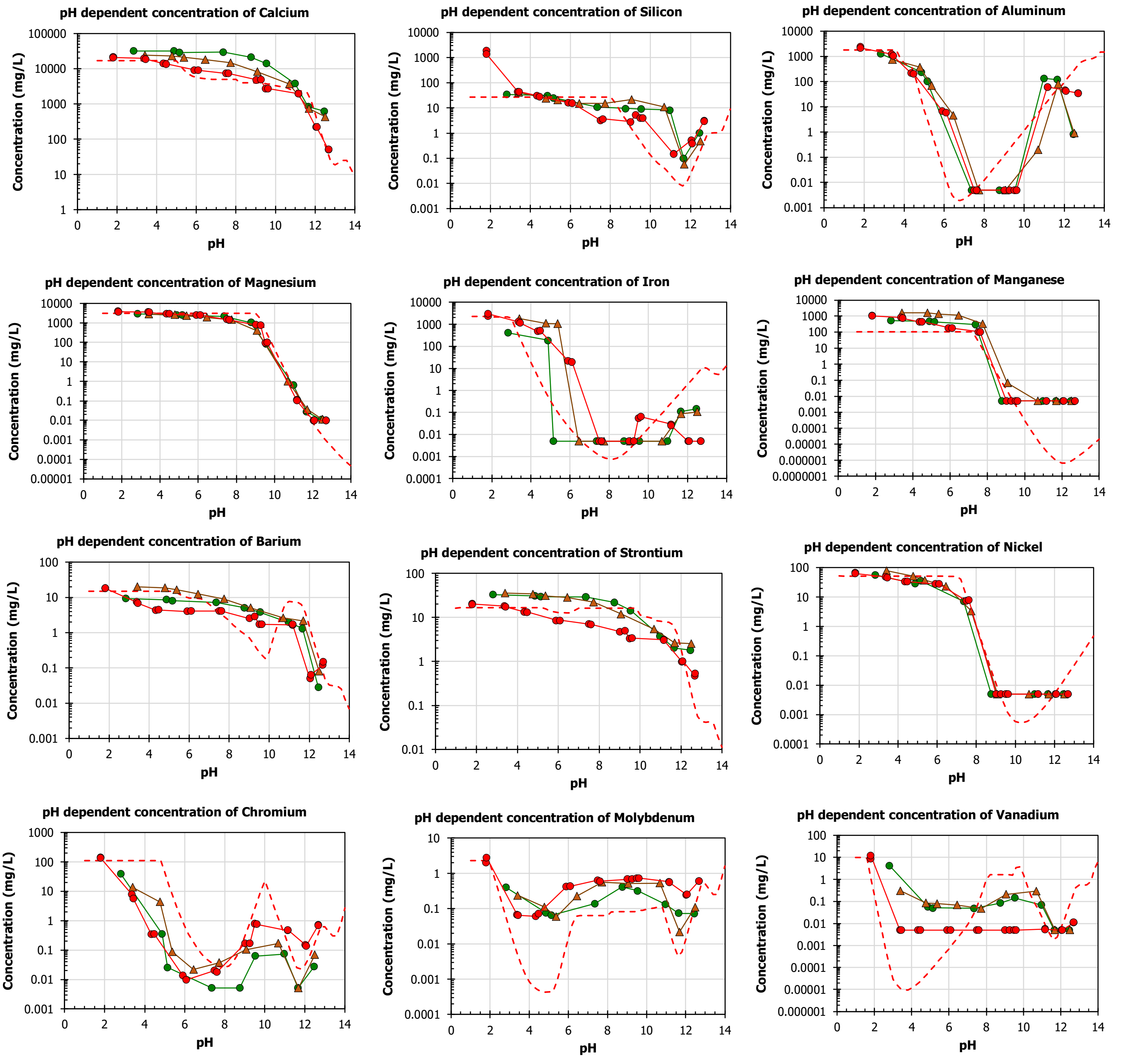

Supplement: Supplementary file 1 [file toxics-10-00010-s001.zip › toxics-1496253-supplementary.pdf]
